# A multi-site data sample for analyzing the online commercial sex ecosystem

**DOI:** 10.1038/s41597-025-04442-w

**Published:** 2025-02-11

**Authors:** Nickolas K. Freeman, Gregory J. Bott, Burcu B. Keskin, Tiffany L. Marcantonio

**Affiliations:** 1 The University of Alabama, Culverhouse College of Business, Tuscaloosa, AL 35487 USA; 2 The University of Alabama, College of Human Environmental Sciences, Tuscaloosa, AL 35487 USA

**Keywords:** Society, Research data

## Abstract

Online sex advertisements (sex ads) have been linked to many U.S. sex trafficking cases. However, since the closure of the dominant website, Backpage.com (Backpage), many competing sites have emerged that are hosted in countries where U.S. law enforcement organizations have no jurisdiction. Although the online ecosystem has changed significantly, very little research uses data from sites other than Backpage, and even less uses data from multiple sites. This paper presents an anonymized dataset derived from the text and image artifacts of more than 10 million sex ads. By making this dataset publicly available, we aim to reduce barriers to entry for researchers interested in conducting data-driven counter-trafficking research. The dataset can be used to test hypotheses related to sex ads and intersite connectivity, understand the posting processes employed by prominent sites in the current online sex ad ecosystem, and develop multidisciplinary approaches for estimating ad legitimacy. Progress in any of these areas can result in potentially lifesaving interventions for ST victims.

## Background & Summary

Sex trafficking (ST) is a form of human trafficking that refers to the use of force, fraud, or coercion to make another individual perform a commercial sex act. In recent years, the number of online escort sites has dramatically increased. Not all escorts are sex trafficking victims. Some are in the business voluntarily, some are fraudulent (falsely advertised to collect deposits without providing service), and based on numerous ST court cases in the U.S., some are sex trafficking victims. However, since the closure of the prominent online sex ad site, Backpage, the online ecosystem of sex ad sites has changed substantially, with many new sites emerging to fill the void. Many of these new sites are hosted in locations where U.S. law enforcement agencies have no jurisdiction. Although some recent studies (^1–4^) consider how sex ads can yield insights into ST, very few acknowledge the data-related challenges that exist in the new sex ad ecosystem and nearly all studies are limited to ads collected from a single site.

One of the first research efforts based on Backpage data showed that the number of sex ads dramatically increased leading up to Super Bowl XLV in Dallas, Texas^5^. Using phone numbers and trafficking indicators published by the Polaris Project and the United Nations Office on Drugs and Crime to link ads, another research effort identified ads likely to be associated with ST^6^. One study^7^ used a specialized machine learning approach that leveraged weak textual features (e.g., age, location, post subject line) to bridge gaps in strong textual features (e.g., phone number) and link ads from the same provider across different phone numbers that might otherwise appear as distinct individuals. Another study^8^ used 600 Backpage ads to develop and test an ST index that combines the features of ethnicity, a legitimate commercial facade, ads posted by others, shared management, movement from another location, and restricted travel (e.g., in-calls only). Again, shared phone numbers were used as a linking mechanism, as well as shared ad identification numbers (ad ID). We are only aware of one project that used sex ad data from multiple sites^9^. This paper used a dataset of approximately 2,000 ads from four sites to investigate recruitment-to-sales pathways across sites.

Prior literature indicates that while many researchers have used sex ads as data inputs for research:

- very few utilize data from more than one site and do not address the presence of scam ads comprehensively,
- data for most existing research was obtained from the now-defunct escort site Backpage.com,
- most research utilizes only phone numbers for identifying individuals, which can be problematic due to the ease of creating new numbers using Voice over Internet Protocol (VoIP),
- the scope and size of the data used is very small (often less than 10,000 ads, some less than 1,000–the present study offers 10 million),
- consequently, very little is understood about how active sites operate and overlap, and
- little is known regarding how the illicit crimes facilitated by sex ads connect to other illicit activities.

The availability of an extensive and complete dataset from nine different websites:

1. enables researchers to better understand the current online sex ad marketplace and its interconnectivity to detect, disrupt, and dismantle the illicit networks hidden therein,
2. facilitates the development and testing of ground-breaking techniques for working with ad data that will improve the quality and validity of insights and policies based on it in the future,
3. lowers barriers to entry for researchers interested in data-driven counter-trafficking research, increasing the diversity of perspectives surrounding this issue in the research literature and
4. provides valuable insights to non-profit organizations and law enforcement in the counter-trafficking domain.

## Methods

### Data Collection Process

**Fig. 1:**
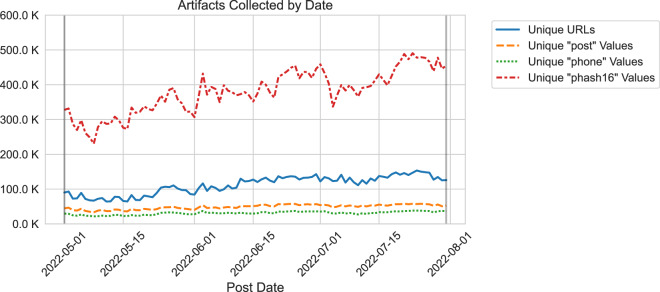
Artifacts Collected by Date.

Sex ad sites deliver high volumes of text and image data to consumers daily. Every day, our data collection process acquires more than 100,000 publicly available ads and references to nearly 400,000 publicly available images, on average, from the top 15 most relevant sex trafficking websites based on volume and feedback from our law enforcement and non-profit partner organizations. Figure 1 shows the number of unique URLs collected per day along with the unique number of *phash16*, *post*, and *phone* artifacts, all of which will be described in subsequent sections, collected over the time horizon from 5/1/2022 to 8/1/2022. Our earlier research^10^ describes our data collection and analysis process in detail. At a high level, our process includes activities for 1) collecting, cleaning, and storing data, 2) linking ads to construct groups of linked data artifacts, 3) constructing visualizations for data groups, and 4) providing these visualizations to LEO partners and non-profit organizations via a password-protected portal. The process described in this paper has assisted law enforcement organizations (LEOs) with identifying and contacting potential ST victims. We use the term ‘potential’ deliberately because the context of a provider is not obvious and takes time to ascertain. During that time, privacy is often afforded to victims for their own protection. Additionally, true sex trafficking victims may vehemently deny they are victimized.

### Linking Images Using Perceptual Hashing

Duplicate sex ads pervade the online ad ecosystem and, if not correctly handled, will skew analyses. For example, do 800 online ads represent 800 unique persons posting a single ad or eight unique persons posting a hundred ads each? Researchers recognize this issue and have commonly used phone numbers to link ads into groups associated with an individual. However, the widespread availability of VoIP services allows providers to change phone numbers easily. Moreover, as Fig. 3 of one study^11^ illustrates, providers often use many phone numbers over time.

Images provide another avenue for deduplication. Although providers typically update post text, ad details, future locations, and phone numbers, they often use a common set of images, which serve as a visual identifier for potential clients. A popular and effective method digital forensics experts use to identify common images is hashing. Although cryptographic hashes, such as MD5 and the SHA family, are often used for strict identification of images, they are ineffective when processing online sex ad images. Online escort sites often watermark, crop, or serve images at different resolutions. In such cases, cryptographic hashes, which are built on a confusion and diffusion paradigm, will produce markedly different hash values, even though the images are very similar. Perceptual hashing techniques can identify similar images that are not identical from the standpoint of a pixel-for-pixel comparison. For example, if two sites serve the same image at different resolutions or with other slight modifications, a perceptual hash can still link the images together. Our data collection process computes the SHA256 (cryptographic) hash and two perceptual hashes based on the methods codified in the ImageHash library provided by Johannes Buchner (see https://github.com/JohannesBuchner/imagehash).

### Standardized Location Analysis

Victim movement provides valuable insights to law enforcement and may indicate ST^12^. Most sex ad sites are laid out in a fashion where individual boards exist for locations, and providers post ads on the boards corresponding to their location. Escorts may also post the same ad in multiple nearby cities and move to the location where demand is highest. Additionally, escorts may post ads in cities they plan to visit soon, indicating future stops on a “tour.” From the perspective of a trafficker, moving victims also offers advantages. First, human trafficking task forces are generally state-funded agencies. Consequently, they focus primarily on their state and engage in limited collaboration with other states. Hence, transporting victims across state lines takes advantage of jurisdiction and coordination limitations. Second, moving victims isolates them and prevents deeper connections with clients who are more likely to report trafficking. Third, transporting victims to different markets can enable traffickers to increase profit by marketing to higher-demand areas. Finally, moving victims frequently keeps them from becoming familiar with their surroundings, making it more challenging to obtain help. Although independent sex workers also choose to travel, groups of escorts traveling together may indicate a pimp or higher levels of organized crime^12^.

Location may also indicate fraud. Fraudulent ads, especially deposit scam ads, are often posted with high volume and across an impossibly large travel area. For example, a sex advertiser indicating availability in California, Texas, Florida, and New York on the same day is not possible for a single provider, which indicates fraud. Since there are no legitimate avenues of recourse for an individual who falls prey to such a scam, the number of such scam ads is significant.

The previous two points describe the importance of analyzing movement when using sex ad data to study ST. However, analyzing movement is not trivial, even if you limit the scope of ad data to a single site. A primary complication is due to how the sites organize their location boards. For example, one popular site provides a board for “Central Texas” but also includes boards for cities considered part of Central Texas (e.g., Austin, Killeen, Bryan-College Station, Waco, etc.). In this example, tracking ads found in Central Texas and one of the cities contained in the Central Texas group is problematic since escorts may post in both the city and the region or location that contains the city. In such a case, should both be counted? On the other hand, if an escort posts in central Texas one day and Austin the next day, did movement occur?

When you expand the set of sites to more than one, tracking providers’ locations becomes more difficult. The increased difficulty results from different sites using different definitions and groupings for locations. Some sites use well-known regional names instead of the individual cities or counties contained within the region. For example, consider the Catskills (i.e., Catskill Mountains) region in New York State, which spans at least four counties. This region is specified as a location on at least one site, whereas others include boards for one or more of the counties in the region. Some sites treat Dallas and Fort Worth as distinct cities, while others combine them into Dallas-Ft. Worth and others have split them into North Dallas, South Dallas, and Ft. Worth. In addition to irregular combinations of cities, single large metropolitan cities like New York provide additional challenges. New York City occupies five boroughs, and escorts may post an ad in the city itself and one or more individual boroughs simultaneously. Further confounding the problem are categories like “New York - Other,” which one of the relevant escort websites uses. Consequently, analyzing movement using data from multiple sites requires location standardization.

Although sites use cities as their primary geographic advertising unit, counties are a more effective analysis unit. Cities suffer from the previously described standardization confusion. Additionally, advertised cities are often close enough for an escort to legitimately work in multiple cities simultaneously. In such cases, treating geographically proximate cities separately can falsely inflate travel estimations. Conversely, using city analysis for large metropolitan cities that span multiple counties (e.g., New York City) may obscure movement. Counties provide a useful level of granularity and consistency. Of course, each proper city has a corresponding county, which was used in the analysis. Specifically, to resolve city and county ambiguity introduced by regions, we mapped regional classifications (e.g., the Mid-Cities in Texas) to the largest city in the region (e.g., Arlington, TX). Then, we mapped it to the corresponding county (e.g., Tarrant County). When performing this mapping, we used the city and county listings listed in version 1.6 of the *Pro* U.S. Cities Database available through simplemaps.com^13^. The benefits of using counties as the measurement unit include county population numbers, law enforcement metrics, LEOs (e.g., country sheriff), and health services, which are often available at the county level. Furthermore, counties such as the Federal Information Processing System (FIPS) are included in census standards. Thus, using the county as the location measurement unit promotes easier data enrichment with additional external sources.

FIPS codes uniquely identify over 3,000 US counties and county equivalents (boroughs, parishes, independent cities). The number of digits in FIPS codes varies depending on the geography described. State codes have two digits (e.g., Alabama = 01), and county codes have five digits, of which the first two are the FIPS code of the state to which the county belongs (e.g., Tuscaloosa, Alabama = 01125). We map all the cities from the nine escort websites to FIPS codes to standardize analysis.

## Data Records

The dataset is available via Harvard Dataverse at 10.7910/DVN/4FMSQD^14^. The authors are part of the Sex Trafficking Analytics for Network Detection and Disruption (STANDD) initiative that is hosted by the Institute of Data and Analytics at The University of Alabama (https://ida.culverhouse.ua.edu/initiatives/human-trafficking/). This paper describes a dataset collected by the STANDD for public use. Our goal in sharing the data is to help researchers interested in pursuing topics related to the commercial sex ad ecosystem and sex trafficking overcome the significant barrier to entry that obtaining data poses. The dataset contains information on all sex ads collected from nine sites during the period of 5/1/2022–8/1/2022 for the continental United States. Table 1 describes the fields included in the data.

**Table 1:** Description of data fields.

Field	Description	Data Type
url	an integer representing the URL associated with the ad.	integer
site	An integer representing the site the data was collected from.	integer
post_date	An integer representing the date the ad was posted in yyyymmdd format	date
post_masked	the raw heading text associated with the ad. Note: Numbers that appear in the text are obfuscated by replacing them with the character *.	string
post_int	an integer representing the raw heading text associated with the ad. Note: all occurrences of a specific text are assigned to the same integer value.	integer
phone_masked	a string representation of the phone number associated with the ad. Note: we were unable to capture the phone number for all ads. In cases where the phone number was not captured, the value is None. Phone numbers are obfuscated by replacing the last four digits with the character *.	text
phone_int	an integer representing the phone number associated with the ad. Note: we were unable to capture the phone number for all ads. In cases where the phone number was not captured, the value is None.	integer
phash16	the perceptual hash for a single image associated with the ad. Note: We use the imagehash (see https://github.com/JohannesBuchner/imagehash) library to compute hash values using the *phash* perceptual hashing technique with arguments hash_size = 16 and highfreq_factor = 1.	text
emojis	a semicolon-separated string containing all unique emojis present in the raw heading text.	text
county_fips	An integer representing the county-level FIPS code corresponding to the location the ad targeted. See https://www.nrcs.usda.gov/wps/portal/nrcs/detail/national/home/?cid=nrcs143_013697 for a list of county-level FIPS codes (last accessed 9/12/2022). Note: all values must be converted to a 5-digit string with zeros added to the left if needed if you want to append other data that uses county FIPS codes. For example, the integer 1010 must be converted to “01010”.	integer
state_fips	An integer representing the state-level FIPS code for the location that the ad targeted. See https://www.nrcs.usda.gov/wps/portal/nrcs/detail/?cid=nrcs143_013696 for a list of state-level FIPS codes (last accessed 9/12/2022).	integer

The data is divided into a set of Parquet files in the online repository. Each Parquet file contains the data for up to 1 Million ads. It was necessary to divide the data to satisfy the size limit on uploaded files. We use the Apache Parquet file format because it 1) is very efficient for storage, 2) is very efficient for loading, and 3) retains data type information in the file, making working with ingested data easier. To help researchers less familiar with the Parquet format get up and running with the data, we provide a Jupyter Notebook that demonstrates how to ingest the data and perform analyses such as assessing the degree of post and phash duplication among sites, understanding frequent emojis, and linking the data in a graph representation. The code is available in the same repository as the data. The code was written using the Anaconda Python distribution, and we provide an environment file to simplify the installation of dependencies for Anaconda users on Windows, Mac, or Linux machines.

Although traffickers and escorts select sites based on the potential to reach the largest audiences possible and are eager for greater promotion and visibility, we avoid assisting sites and traffickers in gaining a wider audience by anonymizing the URLs and site names. To protect the provider (who may be a victim), in addition to withholding the URL and images, we also obfuscate the phone number.

## Technical Validation

Our data collection methods are rigorous, but no method is ever perfect. Due to code-breaking website changes, sustained power outages, and lack of Internet access, the data collection process experienced interruptions. However, because escort sites maintain ad history for days to years, in nearly all instances, we were able to “catch up” on data collection. However, in the busiest cities, it is possible that some ads paged out before resuming data collection. Though rational and largely consistent, mapping regions to counties results in a small loss of geographic granularity. Also, some sites protect contact information behind mechanisms that prevent programmatic extraction. In such cases, we use the value “-1” to indicate the data was inaccessible. Although escort sites contain sex trafficking victims, not all ads represent victims. As stated earlier, a significant number fraudulently collect deposits without providing services, while others are posted voluntarily by individuals in the business exercising some level of agency^15,16^. As with any dataset, we encourage researchers to carefully consider the domain expertise required to properly analyze and interpret the data, especially incorporating the insights and perspectives of ST survivors.

## Usage Notes

### Law Enforcement and Non-Profit Interactions

The primary purpose of our data collection is to make it available to organizations dedicated to ending ST. We have provided data to more than ten such organizations. Broadly, these organizations include task forces designed to reduce rates of ST (e.g., West Alabama Human Trafficking Task Force), individual law enforcement (e.g., local police departments in AL, CO, MS, NY, and Homeland Security), non-profit organizations (NPOs) conducting outreach (e.g., Trafficking Hope, Worthy^2^, Covenant Rescue Group), and rehabilitative NPOs (e.g., The Wellhouse). We hope that the availability of this dataset will help researchers uncover ground-breaking techniques for working with ad data that will improve the quality and validity of insights and policies focused on ST moving forward. Despite several different approaches for linking ads to sex trafficking victims and traffickers^7,8,11,12,17,18^, we still have very few definitive answers. One major barrier to obtaining answers is the inaccessibility of data–especially from the dominant escort site in the United States, which we are providing in the hopes that researchers will make new connections and find new patterns that help strengthen the relationships between sex trafficking and commercial sex ads.

We provide a full, targeted, and unredacted dataset to our NPO outreach partners and witness firsthand how they use it to link escort ads to sex trafficking victims. Using the full set of this data, non-profit volunteers and some former ST victims reach out to individual escorts (who may be voluntary, involuntary, or fraudulently posing as escorts) and provide a “way out” for those who need it. We provide the data in a table format so volunteers do not have to navigate through explicit online sex ads that may re-traumatize them. Further, given our rigorous process to remove scam sex ads from the dataset, volunteers no longer waste valuable time reaching out to false ads. Although all the data are available if the organization requires it, volunteers can be provided with a sanitized and focused list of online escorts, some percentage of whom are ST victims.

Sex ads also provide insights into the ever-changing network and communication of ST^8^ and, broadly, commercial sex work. Having additional insights into the ST world may be particularly beneficial as working directly with people who are ST victims or engaged in commercial sex work may be difficult as this is a hard-to-reach population that is consistently moving. Thus, our ad data may be of value to researchers in social science fields, such as psychology, public health, or communications, who want to assess further how people communicate around ST and commercial sex work–such as emoji use within this area and how this is (or is not) reflective of larger sexual communication patterns.

Regarding policy, this ad data may be helpful for areas that want to address ST within their community. Specifically, ST and, broadly, commercial sex work are behaviors that can occur in response to systematic injustices and disadvantages that certain communities face, such as lack of access to economic growth and development, communities experiencing problems with substance use and mental health concerns, or lack of affordable housing opportunities. If certain communities find they have a higher percentage of sex ads posted than others and thus may have more ST occurring within their area, larger policies could be created to address this. For instance, additional policies could be created to protect those who are ST or engage in commercial sex work so they do not have to fear being arrested for the behavior. Communities could also allocate additional funding to NPOs and task forces that are focused on assisting people with finding job opportunities or stable housing so they need not turn to commercial sex for their livelihood.

Finally, ST or commercial sex work may also be greater in areas that experience higher levels of discrimination against historically marginalized groups, such as people of color^19^. Researchers could combine our sex ad data with larger policy data focused on examining state and county policies focused on protecting the rights of historically marginalized groups to assess if the two are related.

## Data Availability

In addition to data, we provide code demonstrating how to work with the provided data. The code also demonstrates how to perform analyses such as assessing the degree of post and phash duplication among sites, understanding frequent emojis, and linking the data in a graph representation. The code is available via Harvard Dataverse at 10.7910/DVN/4FMSQD^14^. We use the Python programming language for all analyses, and the code is given as a Jupyter Notebook. The first cell in the notebook provides explicit details regarding third-party packages that are needed to replicate the expected analyses and the expected file structure. We use the Anaconda Python distribution (free) and have provided an environment file to simplify the installation of dependencies for Anaconda users using Windows, Mac, or Linux operating systems.
